# Application programming interface guided QA plan generation and analysis automation

**DOI:** 10.1002/acm2.13288

**Published:** 2021-05-26

**Authors:** Matthew C. Schmidt, Caleb A. Raman, Yu Wu, Mahmoud M. Yaqoub, Yao Hao, Rebecca Nichole Mahon, Matthew J. Riblett, Nels C. Knutson, Erno Sajo, Piotr Zygmanski, Marian Jandel, Francisco J. Reynoso, Baozhou Sun

**Affiliations:** ^1^ Department of Radiation Oncology Washington University School of Medicine St. Louis MO USA; ^2^ Department of Physics University of Massachusetts Lowell Lowell MA USA; ^3^ Brigham and Women’s/ Dana Farber Cancer Institute/ Harvard Medical School Boston MA USA

**Keywords:** API, ESAPI, PDSAPI, QA automation, quality assurance

## Abstract

**Purpose:**

Linear accelerator quality assurance (QA) in radiation therapy is a time consuming but fundamental part of ensuring the performance characteristics of radiation delivering machines. The goal of this work is to develop an automated and standardized QA plan generation and analysis system in the Oncology Information System (OIS) to streamline the QA process.

**Methods:**

Automating the QA process includes two software components: the AutoQA Builder to generate daily, monthly, quarterly, and miscellaneous periodic linear accelerator QA plans within the Treatment Planning System (TPS) and the AutoQA Analysis to analyze images collected on the Electronic Portal Imaging Device (EPID) allowing for a rapid analysis of the acquired QA images. To verify the results of the automated QA analysis, results were compared to the current standard for QA assessment for the jaw junction, light‐radiation coincidence, picket fence, and volumetric modulated arc therapy (VMAT) QA plans across three linacs and over a 6‐month period.

**Results:**

The AutoQA Builder application has been utilized clinically 322 times to create QA patients, construct phantom images, and deploy common periodic QA tests across multiple institutions, linear accelerators, and physicists. Comparing the AutoQA Analysis results with our current institutional QA standard the mean difference of the ratio of intensity values within the field‐matched junction and ball‐bearing position detection was 0.012 ± 0.053 (*P* = 0.159) and is 0.011 ± 0.224 mm (*P* = 0.355), respectively. Analysis of VMAT QA plans resulted in a maximum percentage difference of 0.3%.

**Conclusion:**

The automated creation and analysis of quality assurance plans using multiple APIs can be of immediate benefit to linear accelerator quality assurance efficiency and standardization. QA plan creation can be done without following tedious procedures through API assistance, and analysis can be performed inside of the clinical OIS in an automated fashion.

## 1 INTRODUCTION

Quality Assurance (QA) of medical linear accelerators (linac) is a critical responsibility of the qualified medical physicist. The increasing complexity in linac technology warrants standardization in clinically practicable QA. Recommendations to reduce redundancy in testing the parameters characterizing the linac and advise on the frequency at which tests are required have been put forth by many publications, including the Task Group 142 from the American Association of Physicists in Medicine.^1^ Although AAPM TG‐142 recommendations are available since 2009, the machine QA tests are not implemented consistently even within the same institutions.^2^ Efficiency, standardization, and error reduction are a central theme to the development of new QA processes, and the technology has been assisted in developing solutions for safer and more effective QA.^3^, ^4^ These solutions lead to effortless generation of QA plans and more efficient and consistent delivery and analysis of these plans.

The burden of generating QA plans increases under several circumstances: (a) the computational strain of loading and unloading high volume images from daily QA image collection require periodic creation of new QA plans, (b) tedious procedures prohibit the transfer of dynamic multileaf collimator (DMLC) QA plans between QA patients, and (c) manually generated DICOM plans may not be modifiable within the Treatment Planning System (TPS) as the manual modifications made within DICOM may not be reproducible within the limitations of TPS functionality. Extensive mechanisms exist by which to create and execute QA for the assortment of linac components. Simple open field deliveries may be executed in service mode of the linear accelerator, while DMLC fields may require a predefined set of control points prior to QA execution. XML‐based control point definition files allow for the rapid prototyping of QA fields, but require additional software licenses and lack interoperability with analysis tools outside of the linac software environment.^5^, ^6^, ^7^, ^8^ The generation of complex QA plans, such as QA plans for Volumetric Modulated Arc Therapy (VMAT), have been subsidized by vendors and researchers.^9^, ^10^ Plans may also be generated and delivered through the clinical Oncology Information System (OIS). Vendor provided application programming interface (API) features allow for the programmatic generation of QA patients and plans.^11^, ^12^

A linac’s electronic portal imaging device (EPID) demonstrates increased efficiency in high resolution QA measurement by facilitating the acquisition of dosimetric images for patient specific dynamic multileaf collimator (DMLC) QA.^9^, ^13^ Output consistency, dose linearity, and high resolution make the EPID effective in image collection for periodic dosimetry and imaging QA and acceptance testing.^3^, ^14^ Due to the digital image output from the EPID, automated analysis can be achieved with commercial software applications^15^, ^16^ or in‐house software solutions.^17^, ^18^ EPIDs have become the standard acquisition devices for common quality assurance data collection.

Through a combination of API technology, EPID data collection, and custom image analysis software, this study demonstrates an automated quality assurance plan creation and evaluation process that can assist clinical institutions in periodic QA. Clinical TPS write‐enabled scripting is an efficient way in generating plans, especially QA plans of predefined collimation jaw and MLC positions. Delivering plans through the clinical OIS allows for images acquired during plan delivery to be automatically transferred to the clinical TPS database, thus facilitating an efficient means for analysis with API assisted image analysis software.

## 2 MATERIALS AND METHODS

### 2.A Plan creation

**Fig. 1:**
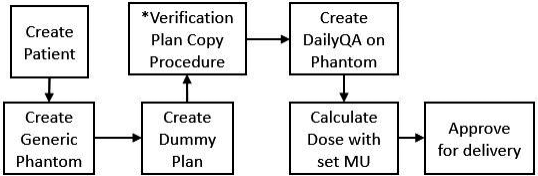
Manual Creation of the Daily quality assurance (QA) plan. *Verification plan copy procedure refers to the use of the verification plan creation functionality within the treatment planning system wherein an image dataset can be transferred from another patient into the current QA patient if that dataset is used to generate a verification plan.

The periodic generation of daily QA plans within our institution’s clinical practice is necessitated by a high volume of patient images, requiring the generation of a new QA patient. When the number of patient images within the OIS reaches a maximum capacity, stress on the database leads to stalled image transfer or even software crashes. The plans could also be delivered through a nonclinical mode (no record‐and‐verify), but the collection and organization of EPID images would be the responsibility of the clinician delivering the QA plan as opposed to the automated transfer and storage of the OIS. A required change in treatment delivery parameters (i.e. treatment machine, energy, or MLC type change) may also require the creation of periodic QA plans. The manual plan creation for daily QA requires several procedural steps (Fig. 1). The time required to complete the daily QA plan creation varies greatly with the experience of the user. The current institutional procedures require monthly and quarterly QA to be delivered through external DICOM files outside of the OIS; any modification required to these DICOM files requires DICOM tag manipulation from custom code.

**Fig. 2:**
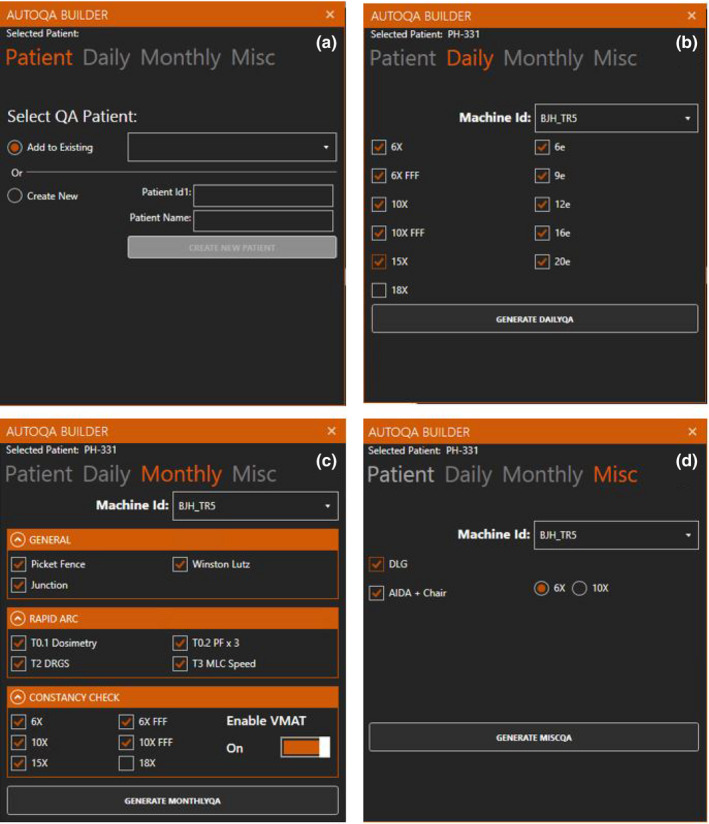
Application for the automated creation of quality assurance (QA) plans. The application facilitates (a) the generation of patients, (b) the creation of daily QA plans, (c) the creation of monthly and quarterly dynamic leaf and volumetric modulated arc therapy QA plans, and (d) additional miscellaneous QA plan generation.

A custom.NET application was developed to expedite the generation of periodic linac QA. The application contains four distinct user‐interaction modules that create QA patients and plans for daily, monthly/quarterly, and miscellaneous QA (Fig. 2). The patient creation is handled through the ARIA Access (AA) Representational State Transfer (REST)^19^ Application Programming Interface (API) wherein the new patient database instance is facilitated through the *CreatePatientRequest* request^20^ (ARIA Access, Version 1.4, Varian Medical Systems, Palo Alto, CA, USA). The image dataset and plan creation are performed using write‐enabled Eclipse Scripting Application Programming Interface (ESAPI) features^21^ (ESAPI, Version 15.6, Varian Medical Systems, Palo Alto, CA). ESAPI enables the creation of the plan, beams, and control points (MLC positioning at beam delivery meterset values and gantry angles) driving the beam delivery. The current version of the API does not allow for setup field creation or electron beam creation; placeholder treatment fields are generated as substitutes for setup and electron beams and must be replaced manually by the user prior to dose calculation. The AA API supports the scheduling of treatment plans for delivery, but the AutoQA Builder leaves the scheduling to the user to allow for a manual review of the plan prior to delivery preparation.

The AutoQA Builder application utilizes a set of JSON formatted treatment field parameter instructions for the creation of DMLC plan creation (i.e. Picket Fence, dosimetric leaf gap (DLG)^22^) and Volumetric Modulated Arc Therapy (VMAT) plan creation.^23^ For fields with less complex MLC motion, such as the Junction and Winston Lutz test, the MLC positions, gantry, collimator, and couch angles are all defined within the source code for the application.^24^, ^25^

### 2.B QA analysis software evaluation

**Fig. 3:**
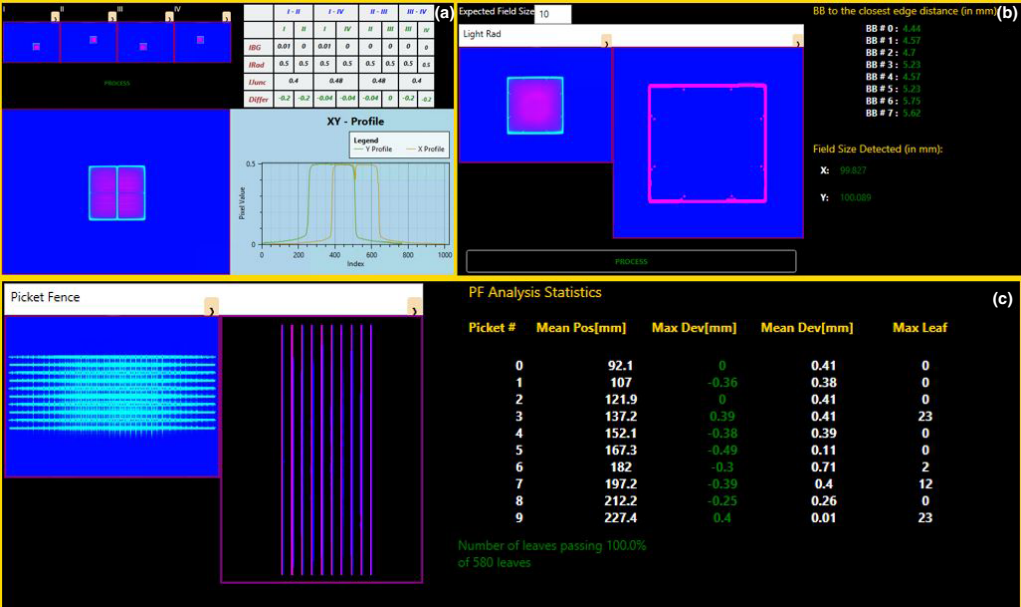
Portal dosimetry scripting application programming interface EPID image QA analysis for (a) Junction, (b) Light‐Rad, and (c) Picket Fence Test.

**Fig. 4:**
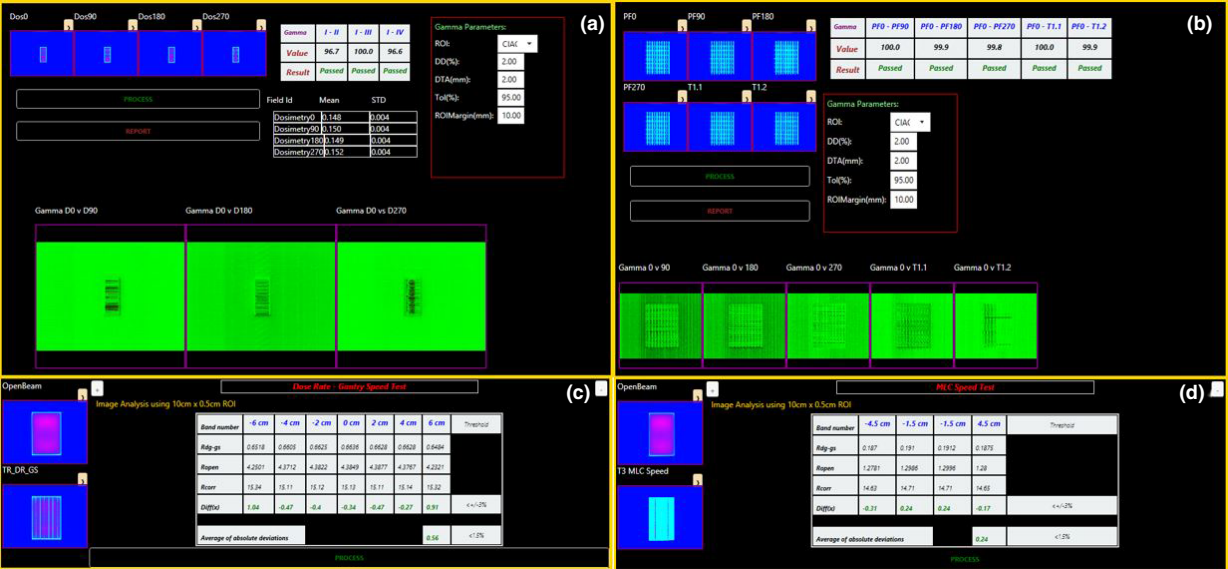
Volumetric modulated arc therapy (VMAT) quality assurance (QA) Tests consisting of (a) static gantry dosimetry consistency, (b) static gantry and rotating picket fence test consistency, (c) dosimetric consistency with variable dose rate and gantry speed VMAT delivery, and (d) dosimetric consistency with variable multileaf collimator Speed VMAT delivery.

With the AutoQA Builder application, QA plans are delivered directly through the OIS (ARIA for Radiation Oncology, Version 15.6, Varian Medical Systems, Palo Alto, CA, USA). After delivery, the integrated images are immediately available in the Portal Dosimetry (PD) application (Varian Medical Systems, Palo Alto, CA, USA). While PD is generally utilized as a patient‐specific QA platform, access to the dosimetry images through the Portal Dosimetry Scripting API (PDSAPI) allows developers direct access to the image pixel information for development of custom QA analysis programs (PDSAPI Version 15.6, Varian Medical Systems, Palo Alto, CA, USA).^26^ The analysis application, herein named AutoQA Analysis, contains various QA test user interface designs to handle monthly (Fig. 3), quarterly (Fig. 4), and Winston‐Lutz QA tests. A set of images is initially selected from the imaging session, and each image is dragged into the test area by the user for the QA analysis.

All tests were acquired on an EPID and analyzed in the AutoQA Analysis application within the Portal Dosimetry workspace. The junction test utilized OxyPlot, an open‐source plotting library for C#, to visualize the dose within the junctions of jaw delimited fields.^27^ The dose within the match point of the jaws should not exceed ±30% of the dose within the open field portions. The light‐radiation field coincidence test^28^, ^29^ utilizes a phantom with ball‐bearing (BB) marker placements at known positions relative to field isocenter.^30^ The analysis of this test required the AForge.NET open‐source C# library, specifically the *SimpleShapeChecker* class that allows for an object within the image—specified by a contrast boundary with its surroundings—to be fit into an assumed shape (a circle).^31^ The picket fence analysis extracted PDSAPI expected MLC positions. The baseline center gap of the expected positions of an MLC pair were compared to the maximum intensity of image profiles through the center of each MLC leaf pair for all MLC in the image.^32^

The quarterly VMAT QA fields consist of static gantry and rotating fields and is based on the RapidArc QA Test Procedures (Varian Medical Systems, Palo Alto, CA, USA).^10^ The first set of four images deliver a sliding window field at cardinal gantry angles, while the second delivers an MLC pattern similar to a picket‐fence for cardinal gantry angles, a rotating gantry image, and a rotating gantry image with MLC position errors introduced into the picket fence. The analysis of these images is performed by internal PDSAPI methods to calculate the 2‐dimensional gamma evaluation using the image at 0‐degree gantry angle as a reference image and the other images for comparison.^33^ The quarterly VMAT QA fields for variable dose rate and gantry speed (T2) and MLC speed (T3) were analyzed automatically by extracting the mean pixel value from regions of interest (ROI) within the image bands [R, Eq (1)] and obtaining a corrected reading by normalizing the mean value within the ROI to an open field delivery [R_Open_, Eq. (1)] to remove beam shape discrepancies. The corrected reading is then converted to a percentage difference from each band’s corrected reading. The result is then the average of the percentage differences of each band [Diff_ABS_, Eq (1)]. Images with similar mean dose in each ROI band (±1.5% tolerance) would be considered passing as per vendor documentation.^10^

(1) $$Diff_{\mathrm{ABS}}\;=\;\frac{1}{N}\left(\sum_{b=1}^{N}\frac{\frac{R}{R_{\mathrm{Open}}}}{\frac{1}{N}\sum_{b=1}^{N}\frac{R}{R_{\mathrm{Open}}}}*100‐100\right).$$

**Fig. 5:**
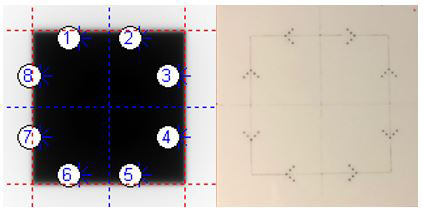
Ball‐bearing placement (left) for paired comparison of light‐radiation coincidence phantom (right).

In validating the output of the AutoQA Analysis application, the monthly QA fields are compared to the current standard of EPID‐QA analysis, DoseLab (Version 6.80, Varian Medical Systems, Palo Alto, CA) over a trial period of 6 months acquired on three different linacs (two TrueBeam and one C‐Series). The calculation of the junction test was performed utilizing the following equation from image profiles gathered with the automated PDSAPI methods and manually by inspecting the profile from within the DoseLab software [Eq. (2)].

(2) $$Junction\;=\;\left(\frac{I_{\mathrm{junc}}‐I_{\mathrm{BG}}}{I_{\mathrm{rad}}‐I_{\mathrm{BG}}}\right)‐1,$$

where $I_{\mathrm{junc}}$, $I_{\mathrm{BG}}$, and $I_{\mathrm{rad}}$ are the intensity values at the junction between the fields, the background reading, and the reading inside the field respectively. The test is considered passing if *Junction* is within ±0.3 (a dimensionless tolerance representing a 30% change from inner field intensity). Light‐radiation coincidence in DoseLab is performed with the user selecting the location of the radio‐opaque makers on a light‐radiation phantom. Therefore, any results from that test will read PDSAPI compared with manually selected positions of the BB markers. The BBs are numbered one (1) through eight (8) where the first BB is the patient right gantry side measuring the distance from the Y2 jaw and BB number increases clockwise around the phantom (Fig. 5). Inner‐most BBs are expected at 5mm from the radiation field edge.

Additional tests are performed to assess the sensitivity of the QA analysis to intentional errors in jaw positioning, MLC positioning, and light to radiation field phantom positioning. The junction test was performed with intentional gaps of 2 mm and 4 mm placed in between the collimator junctions as well as 2 mm and 4 mm overlaps between the jaws. The light‐radiation coincidence phantom was setup at varying SSDs of (95, 97.5, 99, 100, 101, 102.5, and 105 cm) prior to image acquisition. Contingency tables are then constructed between PDSAPI and DoseLab using the passing and failing values of each test for the junction and light‐radiation coincidence tests. Intentional errors of 2, 1.5, 1, and 0.5 mm were introduced to the leaf positions of the picket fence test in both directions. This was then compared to a nominal planned MLC position without intentional errors.

The quarterly VMAT QA analyses are compared to a manual analysis of the same fields with vendor‐provided excel spreadsheet calculations on two TrueBeam linear accelerators (the third linac cannot perform VMAT QA). The daily QA plans consist of mostly open‐field delivery on a daily QA phantom and image setup consistency, and thus far all Daily QA3 (Sun Nuclear Corporation, Melbourne, FL, USA) results have been within expected daily fluctuations and do not warrant further analysis. Validation of the Winston‐Lutz test creation and evaluation was performed prior to this work.^18^ The *P*‐values are obtained through a two‐tailed, paired Wilcoxon Signed Rank Test wherein the significance threshold of (0.05) represents a statistically significant difference in the reported outcome of the test between DoseLab and PDSAPI.

## 3 RESULTS

**Fig. 6:**
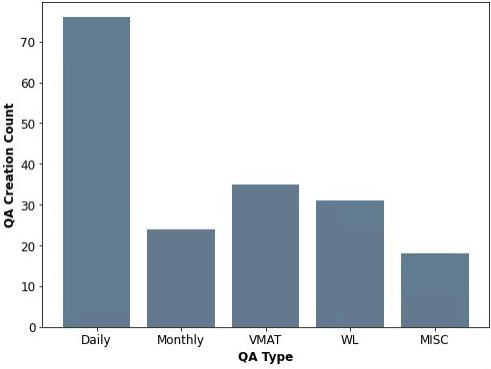
QA plan creation count of varying types using the AutoQA Builder application.

The AutoQA Builder has been executed a total of 322 times in the 14 months since its deployment (Fig 6). 184 times the application was utilized to create a QA plan and the other modification instances were used in conjunction with QA plan creation to generate or modify necessary phantom structures for imaging QA. Daily QA creation is most prevalent with 76 creation instances, an unsurprising result given our departmental policy of quarterly daily QA plan creation. “MISC” covers plans that were generated with the AutoQA Builder for linear accelerator commissioning, Portal Dose Image Prediction (PDIP) algorithm verification, or PD constancy checks.

**Fig. 7:**
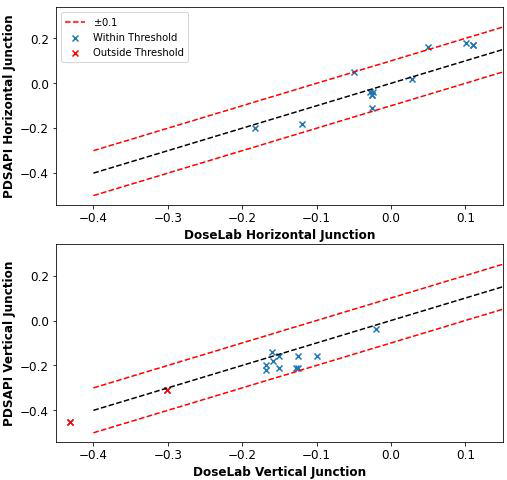
Junction test results acquired manually from DoseLab off‐axis profiles (X‐axis) and reported by the portal dosimetry scripting application programming interface Monthly QA module (Y‐axis) for horizontal (x‐jaw) and vertical (y‐jaw) junctions.

The correlation between the junction test results evaluated in DoseLab and PDSAPI are displayed in Fig. 7. Analysis points that were acquired by applying Eq. (2) showed agreement between DoseLab and PDSAPI within ±0.1 (10% of inner radiation intensity value) for all analysis points except one horizontal junction intensity difference at 0.11 (11% from the inner radiation field intensity value) difference. The mean difference of the PDSAPI junction results subtracted from the DoseLab junction results was 0.012 ± 0.053 (*P* = 0.159).

**Fig. 8:**
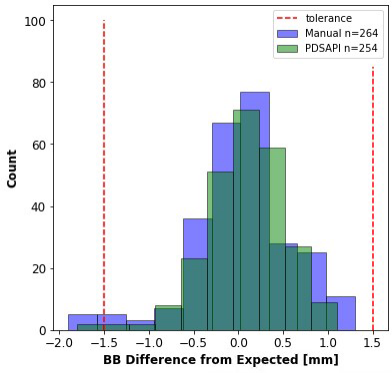
Manually selected (DoseLab) and portal dosimetry scripting application programming interface determined BB position in light‐radiation coincidence phantom relative to the expected position of 5 mm.

Figure 8 shows the distribution of the position relative to the anticipated BB position (5mm from the radiation field edge) wherein the BB placement contain five failing results (n = 264) and two failing results (n = 254) for the manually selected (DoseLab) and PDSAPI determined BBs. Note that the number of detected markers is greater for the manually selected QA results because the user may still be able to determine BB locations that are in near proximity to the edge of the field, due to phantom or jaw misalignment, and the PDSAPI automated detection is unable to determine the BB from the edge of the field.

Numerical analysis of the BB placement in this test was performed (Table 1). To determine the accuracy of BB detection, the absolute value of the deviation of the BB placement from the expected position was compared for BB1–BB8 by subtracting the PDSAPI detected marker position from the manually determined position. Of the eight detected markers, three differences are considered statistically significant (BB 2, 6, and 8). Positive mean values would correspond to the PDSAPI method being closer to the expected position than manual detection.

**Table 1:** Light‐radiation coincidence phantom ball‐bearing (BB) detection deviation of each paired BB set.

BB #	Mean [mm] ± STD (*P*‐value)	N
1	−0.004 ± 0.180 (0.829)	27
2	−0.085 ± 0.189 (0.022)	27
3	−0.042 ± 0.166 (0.161)	33
4	−0.006 ± 0.239 (0.727)	33
5	−0.002 ± 0.157 (0.598)	33
6	0.130 ± 0.251 (0.005)	33
7	0.006 ± 0.268 (0.343)	33
8	0.068 ± 0.236 (0.027)	33
Total	0.011 ± 0.224 (0.355)	252

**Fig. 9:**
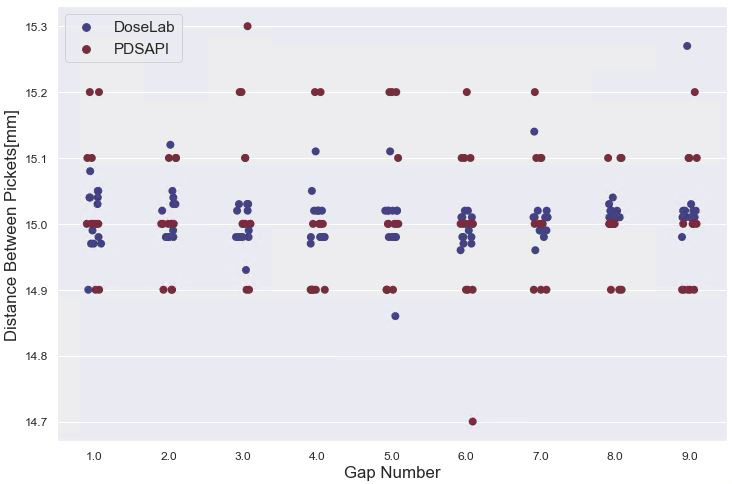
Distance between each high intensity stripe for each picket in the picket fence test.

Figure 9 shows the distance between each picket, grouped by the gap number (i.e. gap number 1 is between picket 1 and 2). The expected distance between pickets is 15mm. For the 13 picket fence tests analyzed, the mean difference between the picket positions (DoseLab – AutoQA Analysis) was 0.041 ± 0.470 mm (*P* = 0.954, n = 208). The mean value of the 13 picket fence deliveries’ maximum deviation of a single leaf from the mean position of the Lorentzian fit (DoseLab) or the expected leaf position from the delivered field’s control point information (PDSAPI) are 0.141 ± 0.163 mm and 0.105 ± 0.485 mm for DoseLab and PDSAPI respectively (*P* = 0.839, n = 13).

The tolerances for VMAT QA are for each normalized band to be within ±1.5% of the average of all normalized bands. Table 2 shows the calculated results—the average of each corrected band normalized to the average corrected reading—for the T2 and T3 VMAT tests.

**Table 2:** Results of the T2 and T3 vendor provided volumetric modulated arc therapy (VMAT) tests.

Test [Tolerance ±1.5%]	Date	Manual Analysis [%]	PDSAPI [%]
T2	09/02/2020	0.35	0.34
	09/25/2020	0.26	0.25
	11/21/2020	0.53	0.56
	12/20/2020	0.35	0.32
T3	09/02/2020	0.48	0.47
	09/25/2020	0.41	0.40
	11/21/2020	0.23	0.24
	12/20/2020	0.51	0.51

**Fig. 10:**
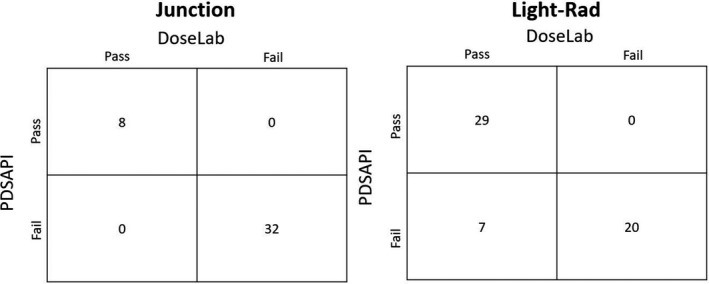
Contingency table between DoseLab manual analysis and portal dosimetry scripting application programming interface for junction tests with intentional gaps and overlaps placed in the field and light‐radiation coincidence tests with varying SSD setup.

To test the sensitivity of each QA analysis application to intentional errors in the delivery of the quality assurance plans, images were acquired with improper geometry and/or phantom placement. The junction test was compared against the DoseLab manually calculated junction intensity values. The contingency table below shows that all tests that passed the DoseLab analysis also passed the PDSAPI analysis and is similar for all failing tests (Fig 10). The light‐radiation coincidence test had some results (7) that failed in the PDSAPI application, but not in the manual BB selection analysis within DoseLab. This yields a sensitivity of 80.5%.

The impact of intentional errors to the picket fence analysis can be seen in Table 3. The images acquired with intentional errors were imported into the nominal picket fence beam, thereby comparing the detected maximum intensity peaks to the nominal beam MLC control point positions and simulating leaf position errors of varying magnitudes. The percentage of leaves passing is reported as the percentage of leaves within 1 mm of the expected position.

**Table 3:** Mean deviation of maximum intensity profile positions subtracted from expected leaf positions of the quality assurance (QA) field.

Picket #	Leaf Position Offset (mm)								
	−2.0	−1.5	−1.0	−0.5	0.0	0.5	1.0	1.5	2.0
Mean Leaf Deviation (mm)									
1	1.59	1.47	0.78	0.33	−0.21	−0.78	−1.21	−1.95	−2.05
2	1.6	1.16	0.71	0.11	−0.39	−0.99	−1.18	−1.93	−2.37
3	1.54	1.03	0.45	−0.07	−0.39	−1.01	−1.55	−1.98	−2.44
4	1.83	1.16	0.78	0.34	−0.39	−0.78	−1.22	−1.68	−2.34
5	1.57	1.21	0.72	0.05	−0.27	−0.82	−1.34	−1.96	−2.22
6	1.55	1.07	0.45	0.01	−0.39	−1.18	−1.29	−1.96	−2.41
7	1.83	1.2	0.78	0.09	−0.43	−0.78	−1.18	−1.63	−2.28
8	1.59	1.08	0.78	0.14	−0.02	−0.78	−1.28	−1.92	−2.38
9	1.57	1.14	0.53	0.01	−0.38	−0.87	−1.54	−1.96	−2.37
# Passing Leaves [%]	0.0	13.0	100.0	100.0	100.0	73.3	0.0	0.0	0.0

## 4 DISCUSSION

Once a QA plan has been designed, the automated generation of those plans assist in the efficiency and consistency with which the tests can be implemented. This leads to each linac within an institution delivering the same QA fields without the organizational burden of having all fields within the same QA patient. Daily QA plans may be generated periodically to prevent the OIS “slow‐down” that occurs with an abundance of 3D images loading at the treatment machine. Additionally, plans that cannot be modified without custom DICOM manipulation code can be generated with ESAPI assistance and modified to meet the user’s need. For example, if a physicist desired to run the VMAT QA tests with an energy other than the vendor provided energy, an energy change in the treatment plan would prompt a recalculation of the dose prior to approval for delivery. The dose on the vendor provided plans cannot be recalculated as the control point spacing is greater than the maximum allowed spacing for dose calculation within the TPS. By resampling the control points with higher resolution, modifications may be made to the energy, treatment machine, and other field parameters that require recalculation allowing the VMAT QA tests to be repurposed for EPID Dosimetry commissioning with different energies and scheduled across different machines.

Automated image analysis with PDSAPI can also lead to efficiency and quality gains. For instance, when analyzing the junction test, a single junction in the vertical and horizontal directions are extracted and used for the calculation of pixel intensity in the overlapping collimator regions. Through programmatic dose profile extraction, all four jaw positioning junctions can be immediately extracted and analyzed. Similarly, the manual determination of BB marker position leads to user inconsistency. Conversely, the manual detection of BB positioning allows for the user to still perform an analysis even when the BB is relatively close to the edge of the field as seen in the decrease in sensitivity for the light‐radiation coincidence test in Fig. 10. Custom software within the clinical OIS also allows for the independence from third party software.

The histogram mean values within each band of the VMAT delivery is expected to be identical between the manual and automated analysis, but errors in user input, determining the correct histogram size, and mathematical rounding may lead to small discrepancies as seen in Table 2. It should be of note that the static gantry fields for VMAT QA do not possess vendor‐provided analysis guidelines, and therefore an analysis of acceptable tolerance limits for distance‐to‐agreement (DTA) and dose difference (DD) within the gamma analysis should be investigated at each institution. The DTA and DD values should remain conservative to visualize of MLC leaves with errors in position, either intentional or by MLC position fault.

## 5 CONCLUSION

The automated creation and analysis of quality assurance plans using multiple APIs can make an immediate and substantial impact on linear accelerator quality assurance efficiency and standardization. Keeping the QA plans and images in the same OIS from beam generation, through delivery, and to analysis assists in the organization of acquired images for the physicists performing the analysis and for future auditing of quality assurance. With hundreds of QA plans generated and dozens of monthly and quarterly QA plans analyzed with the assistance of APIs at our institution, the QA delivery and analysis landscape is changing to a more streamlined and homogeneous approach.

## AUTHOR CONTRIBUTION

MCS, NCK, BS, and FJR contributed to the initial ideas that lead to the creation and implementation of the methods described in this project. CAM, YW, and MCS contributed to the software development of the AutoQA Builder and AutoQA Analysis applications. RNM, MJR, YH, CAM, and MMY assisted in the validation of the tool against current clinical standards. MCS wrote the initial manuscript. ES, PZ, and MJ assisted in guiding the manuscript for completeness and all authors contributed to the review and revision of the manuscript.

## Data Availability

The data that support the findings of this study are available from the corresponding author upon reasonable request.
